# Predicting coefficient of volume compressibility of fine-grained soils using appropriate soil type and soil state parameters

**DOI:** 10.1038/s41598-025-85345-z

**Published:** 2025-01-11

**Authors:** K. S. Vivekananda, H. B. Nagaraj

**Affiliations:** Department of Civil Engineering, B.M.S. College of Engineering, Bangalore, Karnataka 560019 India

**Keywords:** Volume compressibility, Shrinkage index, Compression index, SPT N_60_-value, Environmental sciences, Engineering

## Abstract

Predicting the coefficient of volume compressibility (*m*_*v*_) would help a field engineer to make a quick estimate of the soil compressibility. The multiple correlations suggested by various researchers as available in the literature indicate the importance of predicting the *m*_*v*_ of soil. The existing correlations as available in literature either use soil state (in the form of SPT *N*-value or unconfined compressive strength or natural water content) or soil type (in the form of plasticity properties). However, using both soil type and soil state parameters in developing any prediction equation would be more reliable. To overcome this limitation of existing correlation equations to predict *m*_*v*_, a simple and reliable method that can be universally applied with appropriate soil type parameter represented by the Shrinkage Index (Liquid Limit-Shrinkage Limit) and soil state parameter represented by standardized SPT *N*_60_ has been proposed. This model is designed to be universally applicable, serving as a valuable tool for practicing engineers and researchers to predict *m*_*v*_.

## Introduction

Compressibility and shear strength characteristics of soils are of primary importance in civil engineering applications^1–4^. Compressibility characteristics can be determined either with the value of the coefficient of volume compressibility (*m*_*v*_) or compression index (*c*_*c*_). To determine either *m*_*v*_ or *c*_*c*_, it is necessary to conduct a routine one-dimensional consolidation test, which is time-consuming. It also requires the expertise of a skilled laboratory technician and an undisturbed sample (which mainly depends on the method of extraction of the sample, the quality of drilling equipment being used, and the skill of the operator). To save time and effort, few researchers in the past have attempted to correlate *m*_*v*_ with index properties^5–15^. Table 1 shows the summary of available correlations for predicting *m*_*v*_.

Understanding the shortcomings of the correlations listed in Table 1 is essential to develop a better prediction model utilizing both soil state and soil type. This is necessitated based on the reported information in the literature that in any attempt to predict the engineering properties, it is very important to consider both the soil state and soil type^16–21^.

The ensuing discussion presents the shortcomings of all the equations to predict *m*_*v*_ listed in Table 1.

**Table 1:** Summary of equations for predicting coefficient of volume compressibility sourced from the literature.

Proposed equation	Parameter representing soil state	Parameter representing soil type	Applicability	Reference
$\:{m}_{v}=\frac{1}{80c}$	*q*_*u*_	-	Japanese Soils	Takenaka ^5^
$\:{m}_{v}=\frac{1}{{f}_{2}N}$	SPT N value	*I*_*P*_	London Clays	Stroud ^6^
$\:{m}_{v}=\frac{1}{{1800\,W}_{SW}+{30\,N}_{SW}}$	*q*_*u*_	-	Japanese Soils	Yao et al. ^7^
$\:{m}_{v}=0.00001{\left({w}_{n}\right)}^{A}$	*w*_*n*_	-	Japanese Soils	Tamura et al. ^9^
$\:{m}_{v}{\sigma\:}_{0}=0.003{\left({C}_{F}\right)}^{0.6}$	*q*_*u*_	*I*_*S*_	Remoulded Soils	Parthasarathy ^10^
$\:{m}_{v}=\frac{1}{52c}$	*q*_*u*_	-	Japanese Soils	Satoh et al. ^11^
$\:{m}_{v}=\alpha\:\beta\:{m}_{v0}$	*q*_*u*_, *w*_*n*_	-	Japanese Soils	Matsubara et al. ^12^
$\:{m}_{v}{\sigma\:}_{v}^{\prime}=0.042+0.00162{I}_{S}$	-	*I*_*S*_	Remoulded Soils	Nagaraj and Sridharan ^8,13^
$\:{m}_{v}=\frac{1}{{fN}_{LCPT}{P}_{a}}$	*N*_*LCPT*_	*I*_*P*_	Chennai Soils	Raghuprasanth et al. ^14^
$\:{m}_{v}=0.006\:({w}_{L}+6.93)$	-	*w*_*L*_	Remoulded Soils	Rehman Zia ur et al. ^15^
$\:{m}_{v}=0.0058\:({I}_{P}+27.2)$	-	*I*_*P*_	Remoulded Soils	Rehman Zia ur et al. ^15^

Takenaka ^5^; Yao et al. ^7^; Satoh et al. ^11^ and Matsubara et al. ^12^ correlated *m*_*v*_ with resistance (parameter indicating soil state) obtained from Swedish Weight Sounding (SWS) test on Japanese soils, which are applicable only in Japan for small buildings and not worldwide. Also, the equations lacked in defining the parameters representing soil type, and hence cannot be holistic for prediction purposes.

Tamura et al. ^9^ correlated *m*_*v*_ with natural water content (parameter indicating soil state) but lacks in defining the parameters representing soil type.

Stroud ^6^ correlated *m*_*v*_ with the plasticity index (*I*_*P*_), which is the numerical difference between liquid limit (*w*_*L*_) and plastic limit (*w*_*P*_). However, volume change can still occur beyond the *w*_*P*_. So, a better parameter representing the soil type to account for the complete range of volume change that can occur from practical considerations in civil engineering practice is needed. Further, the equation suggested, is applicable only for a pressure increment of 100 kN/m^2^ in excess of effective overburden pressure. In actual civil engineering practice, the pressure that the soil may be subjected to may exceed 100 kN/m^2^. Hence, this becomes an additional shortcoming.

Nagaraj ^8^ and; Nagaraj and Sridharan ^13^ correlated *m*_*v*_ with Shrinkage index (*I*_*s*_), which is defined as the numerical difference between liquid limit (*w*_*L*_) and shrinkage limit (*w*_*S*_). The shrinkage limit is a state beyond which further volume change ceases. The consolidation process and shrinkage process can be considered to be similar, in the sense that, in both processes volume change occurs; in the former volume change occurs by external sustained loading, and in the latter, it occurs by internal capillary stresses. Hence, considering volume change from *w*_*L*_ to *w*_*S*_ in the form of *I*_*S*_ is a better parameter to represent soil type for volume change behavior. However, the limitation of the correlation suggested, is that the correlation lacks information on the natural state of soil as it is based on remolded samples.

Parthasarathy ^10^, extending the use of *I*_*S*_ as suggested by Nagaraj ^8^, introduced unconfined compressive strength (*q*_*u*_) as a parameter to represent soil state in developing an equation for predicting *m*_*v*_. Test results carried out on soil samples with different initial conditions, like slurry consolidated, compacted, and saturated swollen samples were made use of in developing the correlation to predict *m*_*v*_. Though the author of the study has considered an appropriate parameter for representing the soil type, the use of remolded samples makes it inadequate in representing the natural state of the soil.

Raghuprasanth et al. ^14^ correlated *m*_*v*_ with the penetration resistance obtained from the light cone penetrometer test relating to *I*_*P*_ as the soil type parameter. This correlation has similar drawbacks to that proposed by Stroud ^6^.

Two correlations were suggested by Rehman Zia ur et al. ^15^, one of which is based on *w*_*L*_ and the other on *I*_*P*_. These correlations, lack information about the natural state of soils and also in the use of appropriate parameters representing soil type.

Based on the above discussion, it is evident that the available correlations to predict *m*_*v*_ cannot be universally adopted. Further, for a meaningful prediction of compressibility for field conditions, it was felt reasonable to relate *m*_*v*_ with some soil parameter determined in the field.

Parthasarathy ^10^, considered undrained strength in the form of *q*_*u*_ as a meaningful parameter representing the soil state. To obtain undrained strength, one has to obtain undisturbed samples. To overcome this limitation, it was felt that the strength of soil from one of the popular in-situ tests, namely the Standard Penetration Test (SPT) could be used in developing the correlation. It is well known that penetration resistance/ blow count SPT N-value has been widely used to predict various engineering properties ^22,23^. SPT N-value and *q*_*u*_, both being a measure of undrained strength, either of them can be considered to represent soil state. Hence, in the present study an attempt has been made to overcome the above-discussed shortcomings to estimate the compressibility of natural soils by using SPT N-value obtained through in-situ testing as a parameter representing the state of soil and *I*_*S*_, representing soil type.

## Materials and methods

### Test location

A sufficient number of undisturbed soil samples (32 in number) were collected from boreholes during field investigations being carried out for various infrastructure projects located within India [Chennai (soil Nos. 1 to 10), Indore (soil Nos. 11 to 14), Kerala (soil Nos.15 to 18) and Odisha (soil Nos. 19 to 22)], and also overseas [Nigeria (soil Nos.23 to 30) and Bangladesh (soil Nos. 31 to 32)].

### Field testing

The collection of undisturbed samples from each of the boreholes under consideration at varying depths was followed by carrying out a Standard Penetration Test (SPT) to record the penetration resistance/blow count (SPT *N*- value) as per IS 2131^24^ in all the field investigations considered in this study. However, the donut hammer was used in India and Bangladesh projects to record the SPT *N*-value, whereas the automating trip hammer was used in Nigeria projects. The differences in energy imparted were suitably standardized as discussed below.

Though the name SPT indicates ‘Standard Penetration Test’, the SPT *N*-value recorded needs to be standardized to account for the variations in hammer energy efficiency depending on the method adopted for dropping the hammer and its dissipation around the sampler. Hence, to ensure accurate interpretation based on SPT *N*-value as initially observed and reported by Ireland et al. ^25^ and; Serota and Lowther ^26^, it is essential to apply corrections to standardize the variations in testing procedures. Field-recorded SPT *N*-values were standardized to 60% of the rated hammer energy as suggested by Eurocode EC7^27^. In addition to energy correction, the *N*-values were standardized by applying corrections for borehole diameter, rod length, hammer efficiency, and sampler type as suggested by Skempton ^28^, and the same has been tabulated in Table 2 as standardized SPT values (*N*_60_).

**Table 2:** Summary of recorded and standardized *N*-values for soil samples obtained from borehole locations of infrastructure projects considered in the present study.

Location	Borehole No.	Soil No.	Depth, (m)	Recorded SPT *N*-value	Correction factor for hammer efficiency	Correction factor for rod length	Correction factor for borehole diameter	Correction factor for sampler setup*	Standardized SPT *N*_60_
Chennai	1	1	12.0	23	0.75	1.00	1.05	1.1	20
	2	2	9.0	14	0.75	1.00	1.05	1.1	12
	3	3	15.0	14	0.75	1.00	1.05	1.1	12
	4	4	6.0	8	0.75	0.95	1.05	1.1	7
	5	5	6.0	13	0.75	0.95	1.05	1.1	11
	6	6	6.0	14	0.75	0.95	1.05	1.1	12
	7	7	12.0	9	0.75	1.00	1.05	1.1	8
	8	8	12.0	8	0.75	1.00	1.05	1.1	7
	9	9	15.0	8	0.75	1.00	1.05	1.1	7
	10	10	18.0	9	0.75	1.00	1.05	1.1	8
Indore	1	11	4.5	14	0.75	0.95	1.05	1.1	12
	2	12	4.5	22	0.75	0.95	1.05	1.1	18
	3	13	4.5	21	0.75	0.95	1.05	1.1	17
	4	14	4.5	19	0.75	0.95	1.05	1.1	16
Kochi	1	15	16	10	0.75	1.00	1.05	1.1	9
	2	16	20.5	12	0.75	1.00	1.05	1.1	10
	3	17	14.5	8	0.75	1.00	1.05	1.1	7
	4	18	14.5	10	0.75	1.00	1.05	1.1	9
Odisha	1	19	2.5	13	0.75	0.85	1.05	1.1	10
	2	20	5.5	16	0.75	0.95	1.05	1.1	13
	3	21	2.5	8	0.75	0.85	1.05	1.1	6
	4	22	2.5	11	0.75	0.85	1.05	1.1	8
Nigeria	1	23	15.0	7	0.98	1.00	1.05	1.1	8
	2	24	18.0	9	0.98	1.00	1.05	1.1	10
	3	25	12.0	12	0.98	1.00	1.05	1.1	14
	4	26	15.0	9	0.98	1.00	1.05	1.1	10
	5	27	12.0	14	0.98	1.00	1.05	1.1	16
	6	28	15.0	12	0.98	1.00	1.05	1.1	14
	7	29	15.0	7	0.98	1.00	1.05	1.1	8
	8	30	18.0	9	0.98	1.00	1.05	1.1	10
Bangladesh	1	31	31.0	22	0.75	1.00	1.05	1.1	19
	2	32	12.5	7	0.75	1.00	1.05	1.1	6

* Sampler liner was not used while performing SPT.

### Laboratory testing

Physical properties such as grain size distribution, plasticity properties, and engineering properties such as one-dimensional consolidation test and unconfined compressive strength tests were carried out on all the undisturbed samples collected from various locations following the procedure as outlined in SP36 (Part 1) ^29^. The test results of physical and engineering properties are respectively tabulated in Tables 3 and 4.

**Table 3:** Summary of physical properties of undisturbed samples collected from various borehole locations of infrastructure projects considered in the present study.

Soil No.	Passing 75 μm, (%)	*w*_*L*,_ (%)	*w*_*P*_, (%)	*w*_*S*_, (%)	*I*_*P*_, (%)	*I*_*S*_, (%)	USCS Classification
1	98	146.9	33.2	19.7	113.7	127.2	CH
2	95	66.0	24.7	14.2	41.3	51.8	CH
3	94	76.5	30.4	16.7	46.1	59.8	CH
4	91	75.4	30.9	18.6	44.5	56.8	CH
5	98	61.0	25.0	13.0	36.0	48.0	CH
6	98	64.0	27.0	11.0	37.0	53.0	CH
7	94	77.0	32.0	17.0	45.0	60.0	CH
8	97	63.0	32.0	18.0	31.0	45.0	MH
9	97	55.0	25.0	14.0	30.0	41.0	CH
10	98	78.0	31.0	24.0	47.0	54.0	CH
11	88	60.9	29.9	2.3	31.0	58.6	CH
12	81	64.4	31.3	5.7	33.1	58.7	CH
13	80	44.2	23.2	4.8	21.0	39.4	CL
14	81	40.6	24.0	8.0	16.6	32.6	CL
15	72	105.4	37.4	16.2	68.0	89.2	CH
16	84	142.5	48.9	18.0	93.6	124.5	CH
17	99	107.7	39.7	17.5	68.0	90.2	CH
18	93	111.8	43.9	21.6	67.9	90.2	CH
19	50	43.0	18.0	14.0	25.0	29.0	SC
20	47	48.0	18.0	15.0	30.0	33.0	SC
21	52	42.0	17.0	14.0	25.0	28.0	SC
22	51	47.0	18.0	17.0	29.0	30.0	SC
23	99	104.0	38.0	17.3	66.0	86.7	CH
24	98	107.0	42.0	19.1	65.0	87.9	CH
25	83	100.0	35.0	16.2	65.0	83.8	CH
26	96	111.0	39.0	17.0	72.0	94.0	CH
27	66	67.0	25.0	14.5	42.0	52.5	CL
28	100	100.0	40.0	19.0	60.0	81.0	CH
29	96	106.0	37.0	16.6	69.0	89.4	CH
30	96	98.0	38.0	18.2	60.0	79.8	CH
31	89	61.2	22.2	14.6	39.0	46.6	CH
32	90	51.1	24.5	17.0	26.6	34.1	CH
CL- Clay of low plasticity, CH – Clay of high plasticity, MH – Silt of high plasticity, SC- Clayey Sand							

**Table 4:** Summary of engineering properties of undisturbed samples collected from various borehole locations of infrastructure projects considered in the present study.

Soil No.	*q*_*u*,_ (kN/m^2^)	*m*_*v*_ (10^− 3^ m^2^/kN)				
		25–50 kN/m^2^	50–100 kN/m^2^	100–200 kN/m^2^	200–400 kN/m^2^	400–800 kN/m^2^
1	268.0	0.519	0.468	0.220	0.122	0.102
2	154.0	0.383	0.356	0.280	0.203	0.134
3	122.0	0.301	0.304	0.248	0.155	0.109
4	66.0	0.617	0.512	0.388	0.380	0.207
5	126.0	0.265	0.352	0.184	0.141	0.093
6	116.0	0.860	0.519	0.272	0.187	0.116
7	76.0	0.302	0.270	0.392	0.394	0.265
8	78.0	0.366	0.583	0.399	0.386	0.265
9	58.0	0.287	0.410	0.322	0.401	0.330
10	74.0	0.209	0.436	0.311	0.379	0.286
11	140.0	0.400	0.375	0.249	0.163	0.105
12	235.0	0.424	0.340	0.265	0.196	0.128
13	213.0	0.399	0.321	0.225	0.146	0.097
14	199.0	0.309	0.250	0.191	0.132	0.080
15	98.0	0.844	0.670	0.401	0.237	0.130
16	127.0	0.593	0.492	0.419	0.380	0.217
17	96.0	1.388	1.029	0.747	0.428	0.283
18	109.0	1.383	0.897	0.577	0.382	0.231
19	114.0	0.255	0.231	0.240	0.219	0.139
20	118.0	0.205	0.206	0.222	0.183	0.116
21	76.0	0.165	0.194	0.160	0.076	0.043
22	112.0	0.225	0.239	0.210	0.166	0.124
23	112.0	0.545	0.565	0.433	0.326	0.245
24	116.5	0.427	0.455	0.380	0.300	0.246
25	150.1	0.376	0.363	0.286	0.275	0.270
26	110.6	0.798	0.634	0.366	0.276	0.275
27	174.0	0.401	0.364	0.255	0.202	0.168
28	154.0	0.510	0.461	0.326	0.288	0.239
29	133.5	0.352	0.352	0.306	0.257	0.274
30	133.5	0.510	0.461	0.326	0.288	0.239
31	231.0	0.260	0.226	0.144	0.104	0.087
32	56.0	0.769	0.576	0.543	0.290	0.158

## Results and discussions

**Fig. 1:**
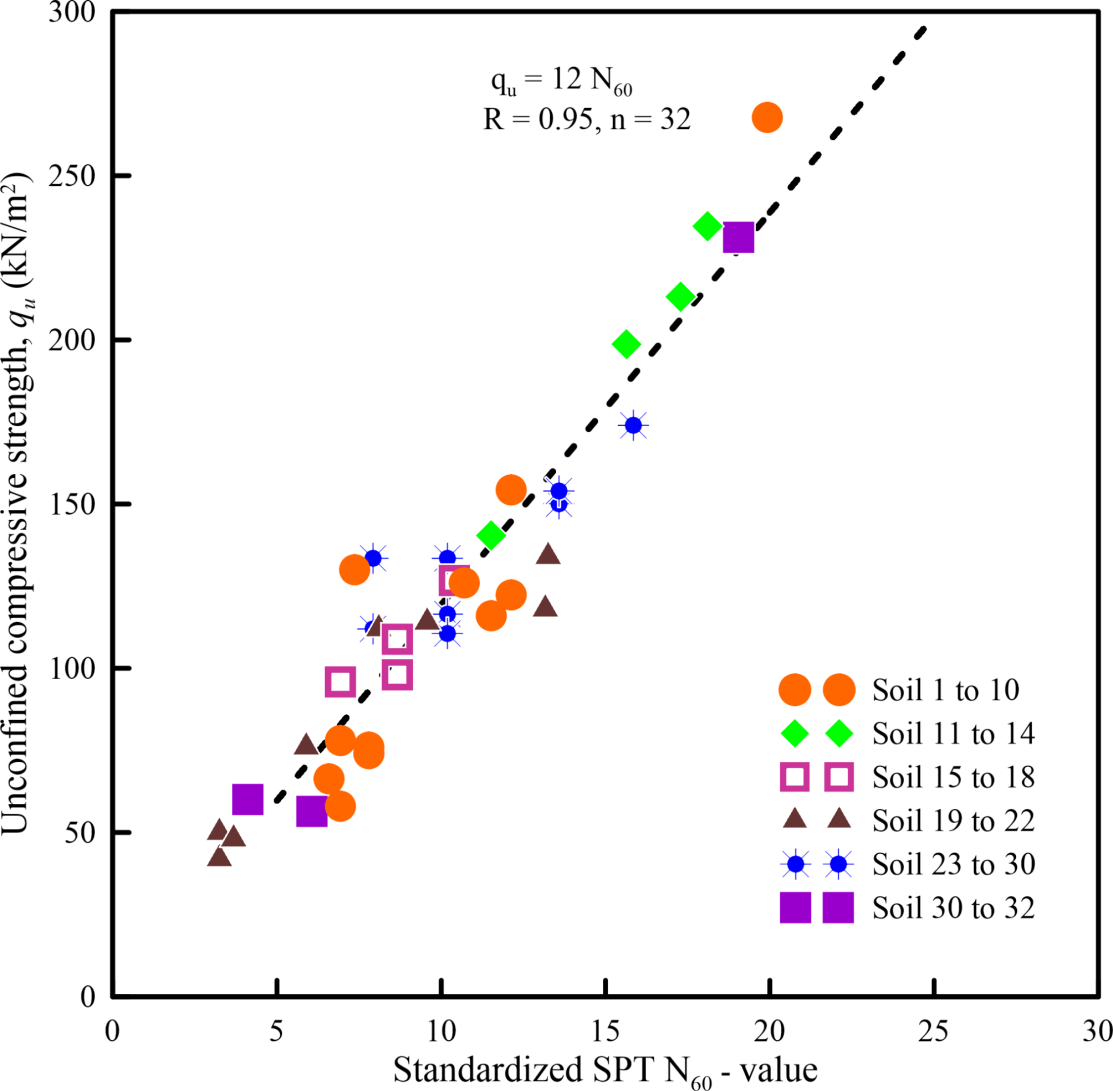
Plot of unconfined compressive strength versus standardized SPT *N*_60_-value for soil samples obtained from borehole locations of infrastructure projects considered in the present study.

Both *q*_*u*_ and SPT *N*-values are measures of the strength characteristics of soil, the former being a direct measurement and the latter being an indirect measurement of strength. Considering this fact, a plot between *q*_*u*_ and standardized SPT *N*-value (*N*_60_) has resulted in a good relationship (*r* = 0.95) as shown in Fig. 1. This indicates that standardized SPT *N*_60_-value can also be a meaningful parameter representing the natural state of the soil, and hence, represent the soil state. Thus, the use of standardized SPT *N*-value will overcome the need for extracting an undisturbed sample to evaluate *q*_*u*_, which can be obtained from Eq. (1).

1 $$\:{q}_{u}=12\:{N}_{60}$$

**Fig. 2:**
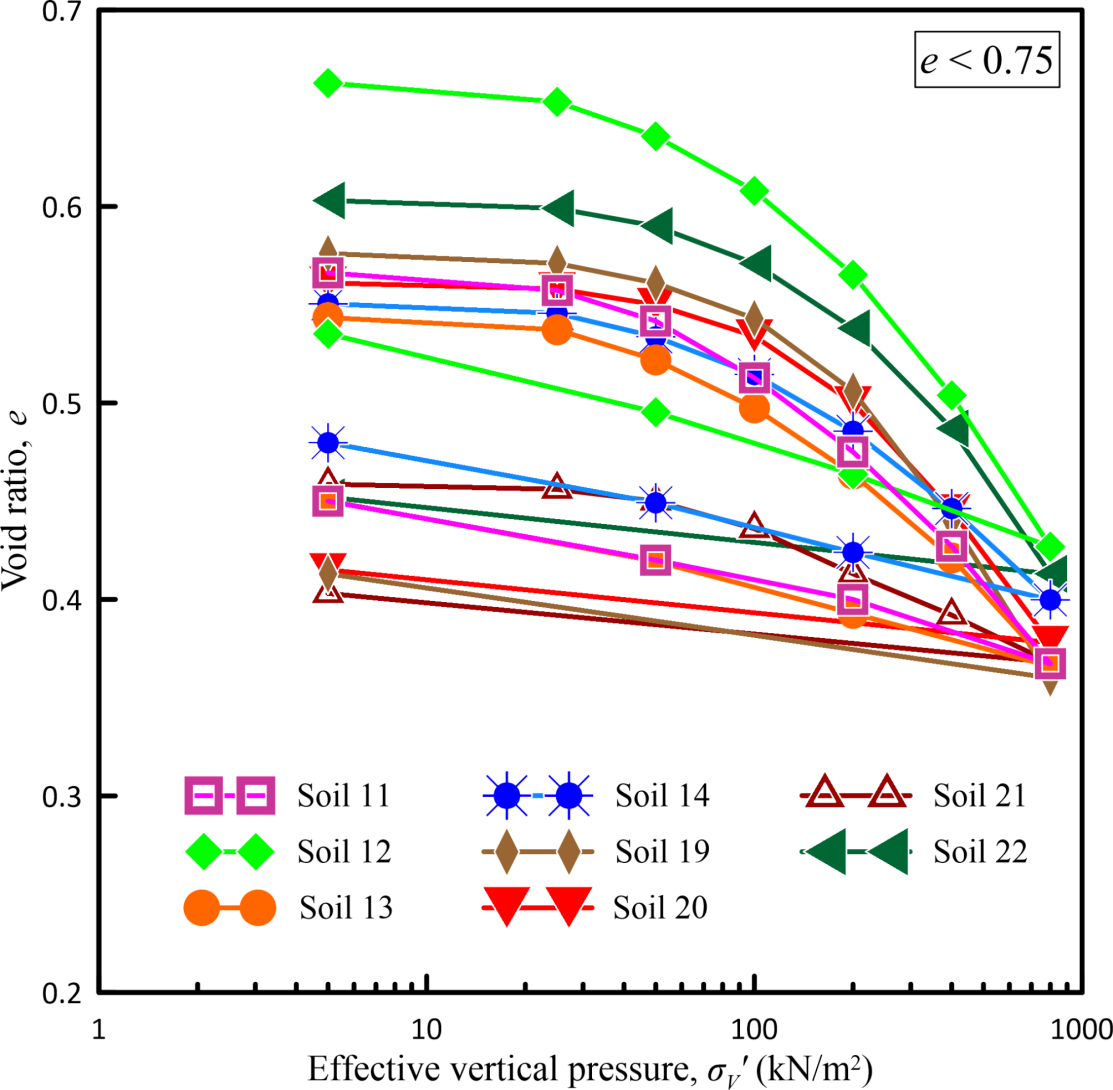
*e*-log *σ*_*v*_ curves for void ratio less than 0.75.

**Fig. 3:**
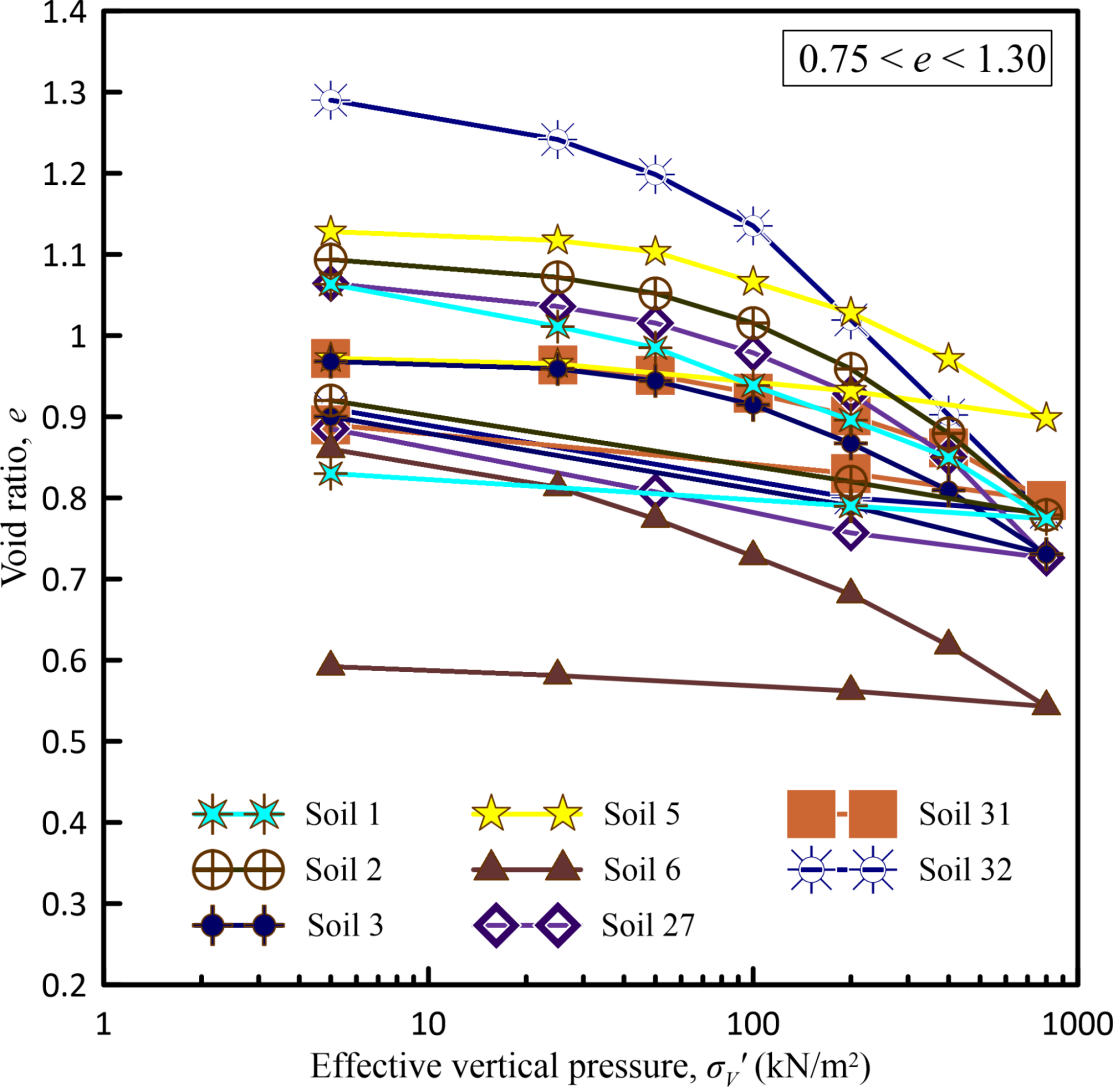
*e*-log *σ*_*v*_ curves for void ratio between 0.75 to 1.30.

**Fig. 4:**
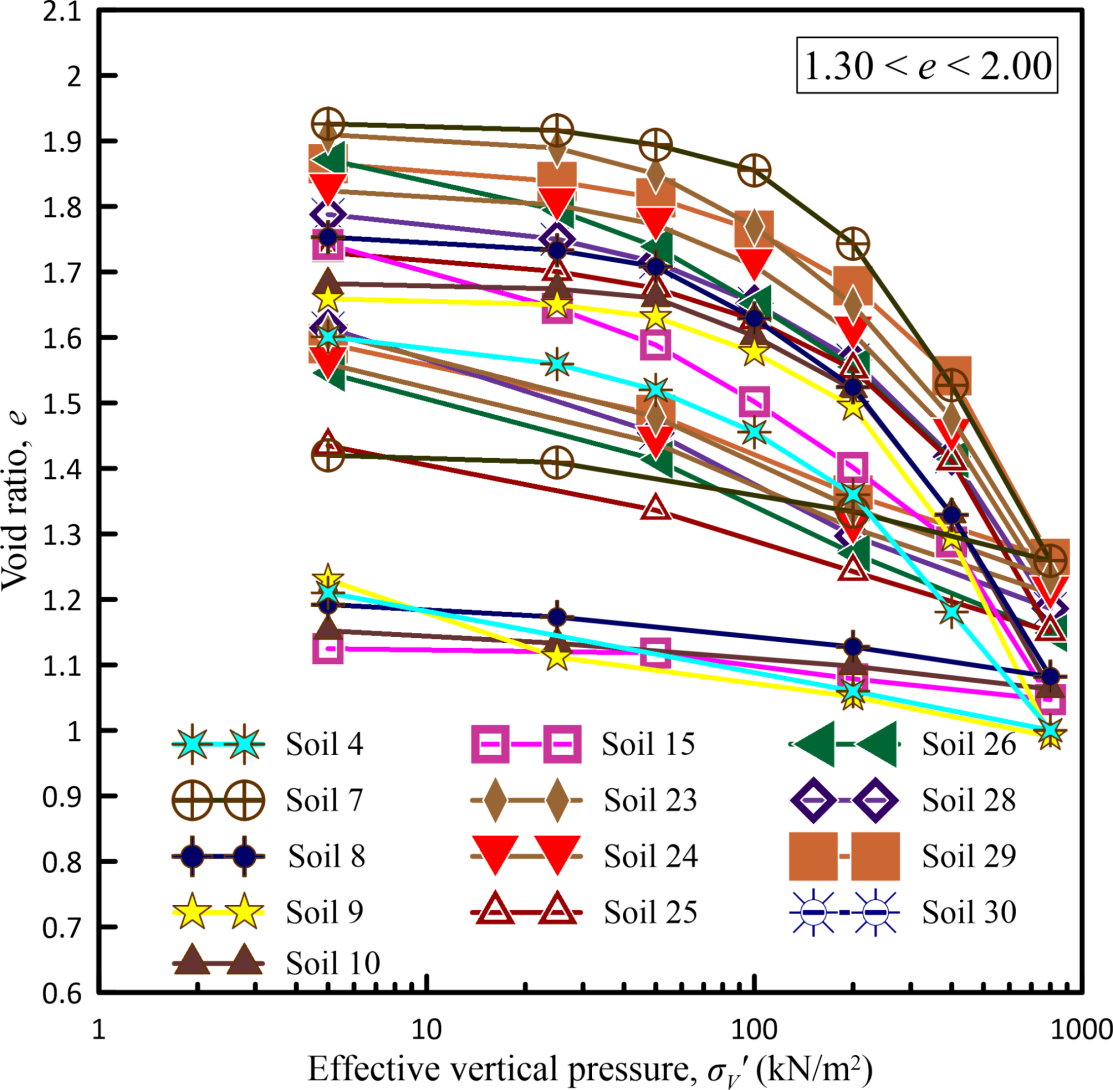
*e*-log *σ*_*v*_′ curves for void ratio between 1.30 to 2.00.

**Fig. 5:**
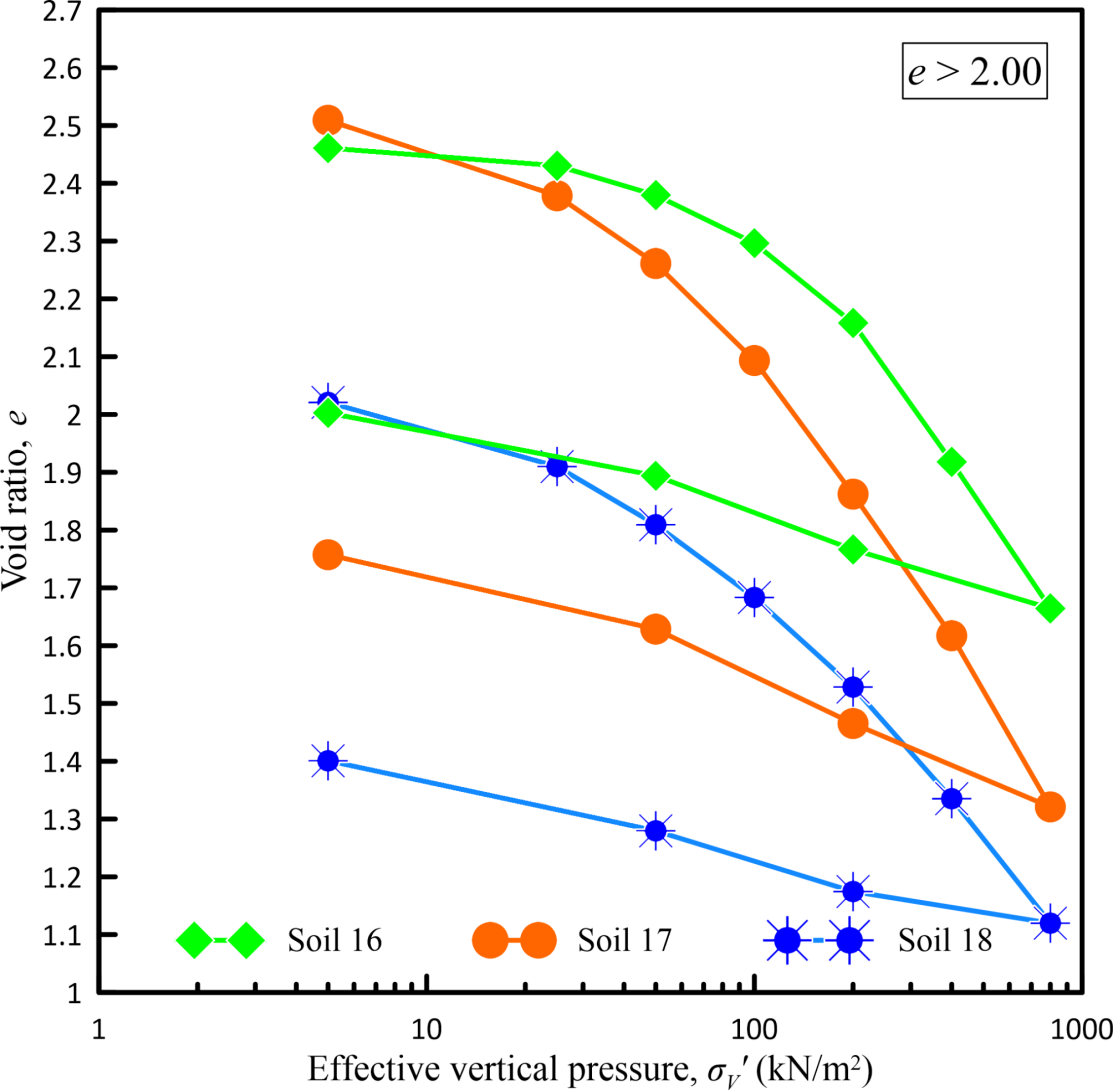
*e*-log *σ*_*v*_′ curves for void ratio greater than 2.00.

In the present study, considering the pressure range up to 800 kN/m^2^ as the maximum pressure that can be experienced in various construction projects like foundations of high-rise buildings, high embankments, or other infrastructure projects, one-dimensional consolidation tests were carried out on all the undisturbed soil samples collected from various borehole locations of infrastructure projects considered in the present study subjecting them to an incremental loading up to a maximum pressure of 800 kN/m^2^. Figures 2, 3, 4 and 5 show *e*-log *σ*_*v*_′ curves for all undisturbed soil samples used in a present study covering a wide range of void ratios from 0.459 to 2.509.

The values of *m*_*v*_ for various pressure ranges, namely, 25–50 kN/m^2^, 50–100 kN/m^2^, 100–200 kN/m^2^, 200–400 kN/m^2^, and 400–800 kN/m^2^ for all soils used in the present study were evaluated and the same have been tabulated in Table 4.

Parthasarathy ^10^, in his study reported that unlike *c*_*c*_, which is a function of only soil type, *m*_*v*_ is a function of both soil state and soil type. Therefore, the author of the study introduced a new term, namely, compression coefficient (*C*_*n*_), which is defined as the ratio of *I*_*S*_ (representing the soil type) to the *q*_*u*_ (representing the soil state) (refer to Eq. (2)). In the present study, as *q*_*u*_ and standardized SPT *N*_60_ have a good relationship, *q*_*u*_ can be determined using *N*_60_ through Eq. (1), thereby eliminating the need for undisturbed sampling to determine *q*_*u*_.

2 $$\:{C}_{n}=\frac{{I}_{S}}{{q}_{u}}$$

**Fig. 6:**
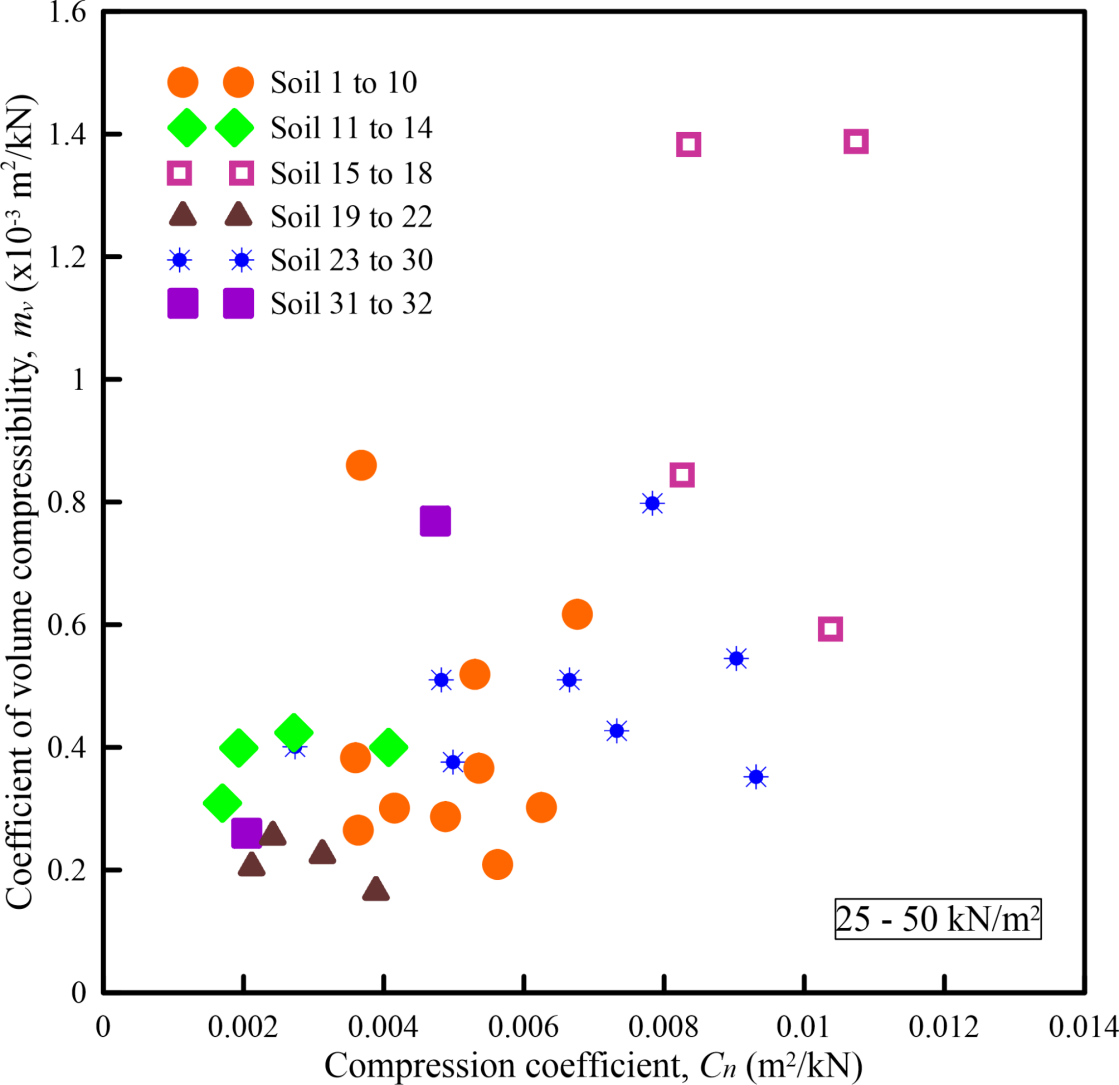
Variation of *m*_*v*_ with *C*_*n*_ for a pressure range of 25–50 kN/m^2^.

**Fig. 7:**
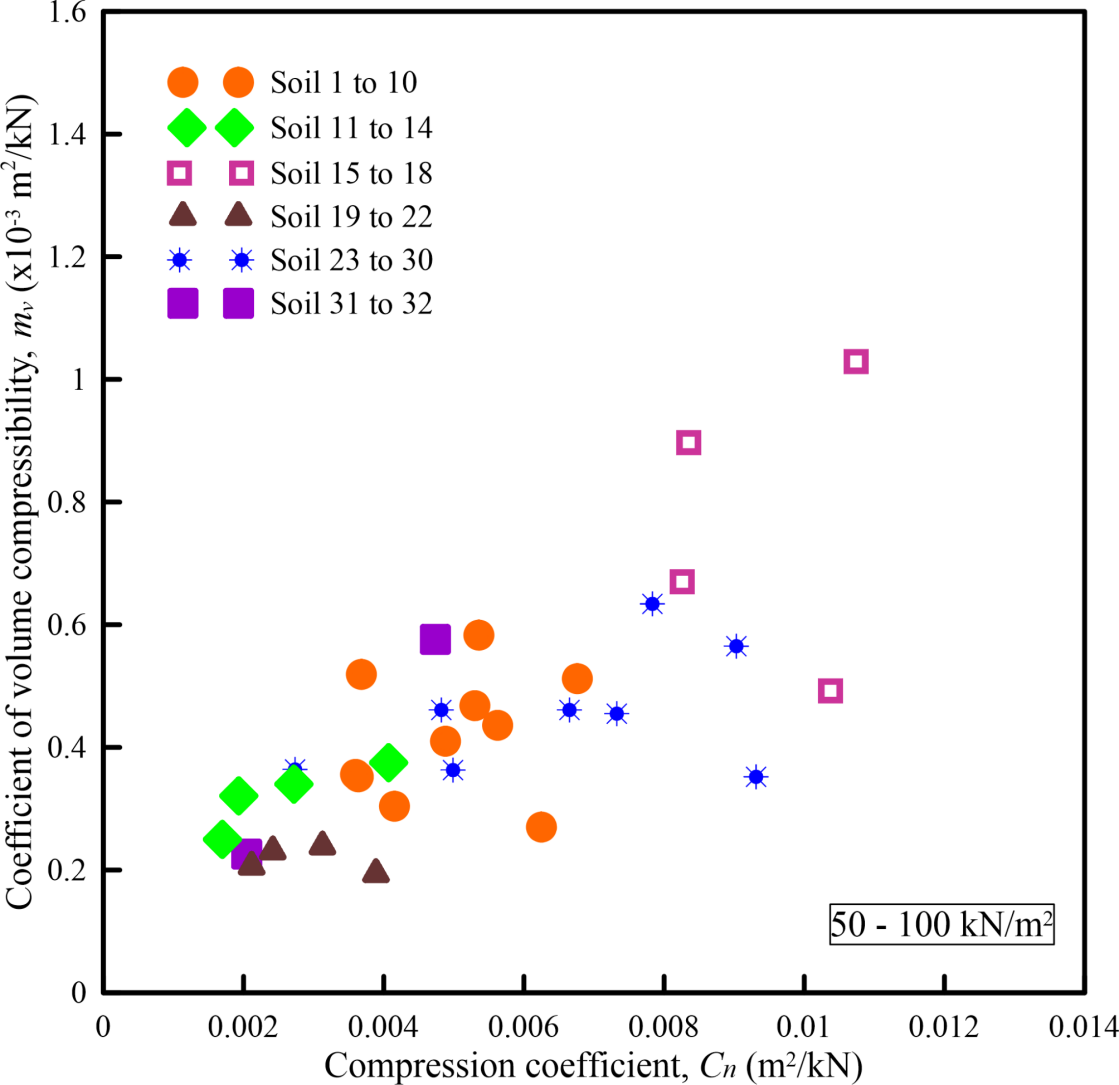
Variation of *m*_*v*_ with *C*_*n*_ for a pressure range of 50–100 kN/m^2^.

**Fig. 8:**
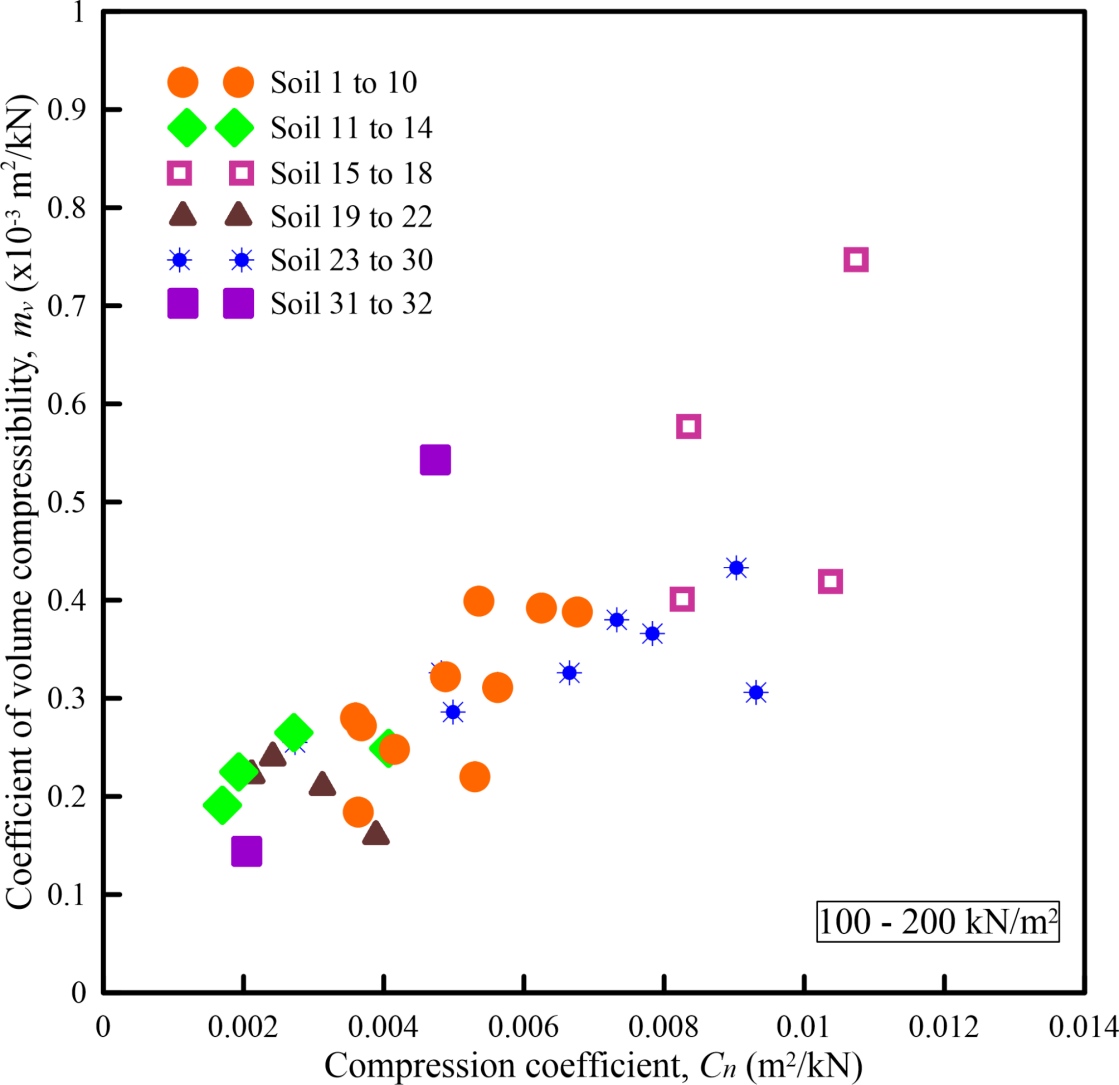
Variation of *m*_*v*_ with *C*_*n*_ for a pressure range of 100–200 kN/m^2^.

**Fig. 9:**
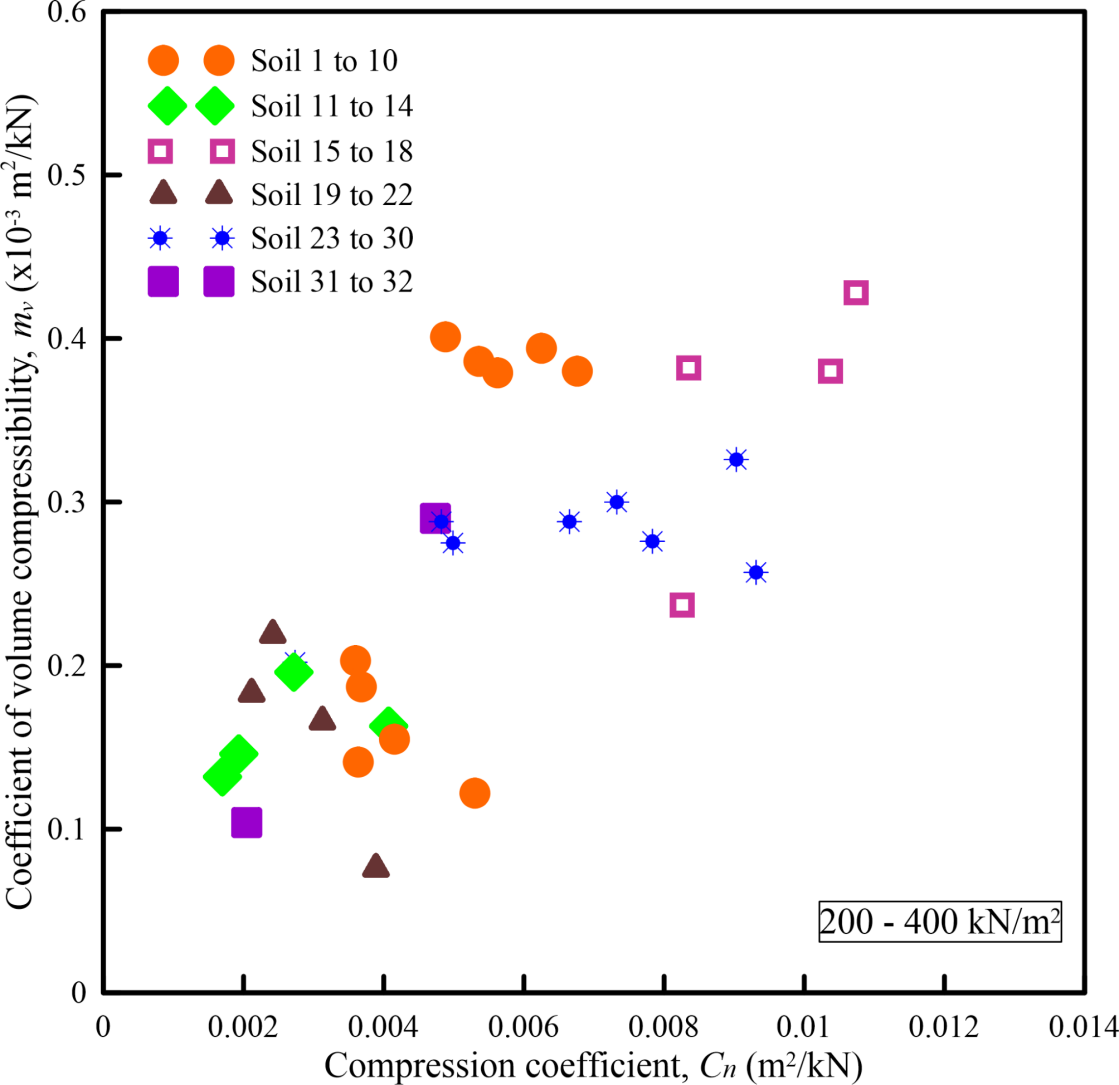
Variation of *m*_*v*_ with *C*_*n*_ for a pressure range of 200–400 kN/m^2^.

**Fig. 10:**
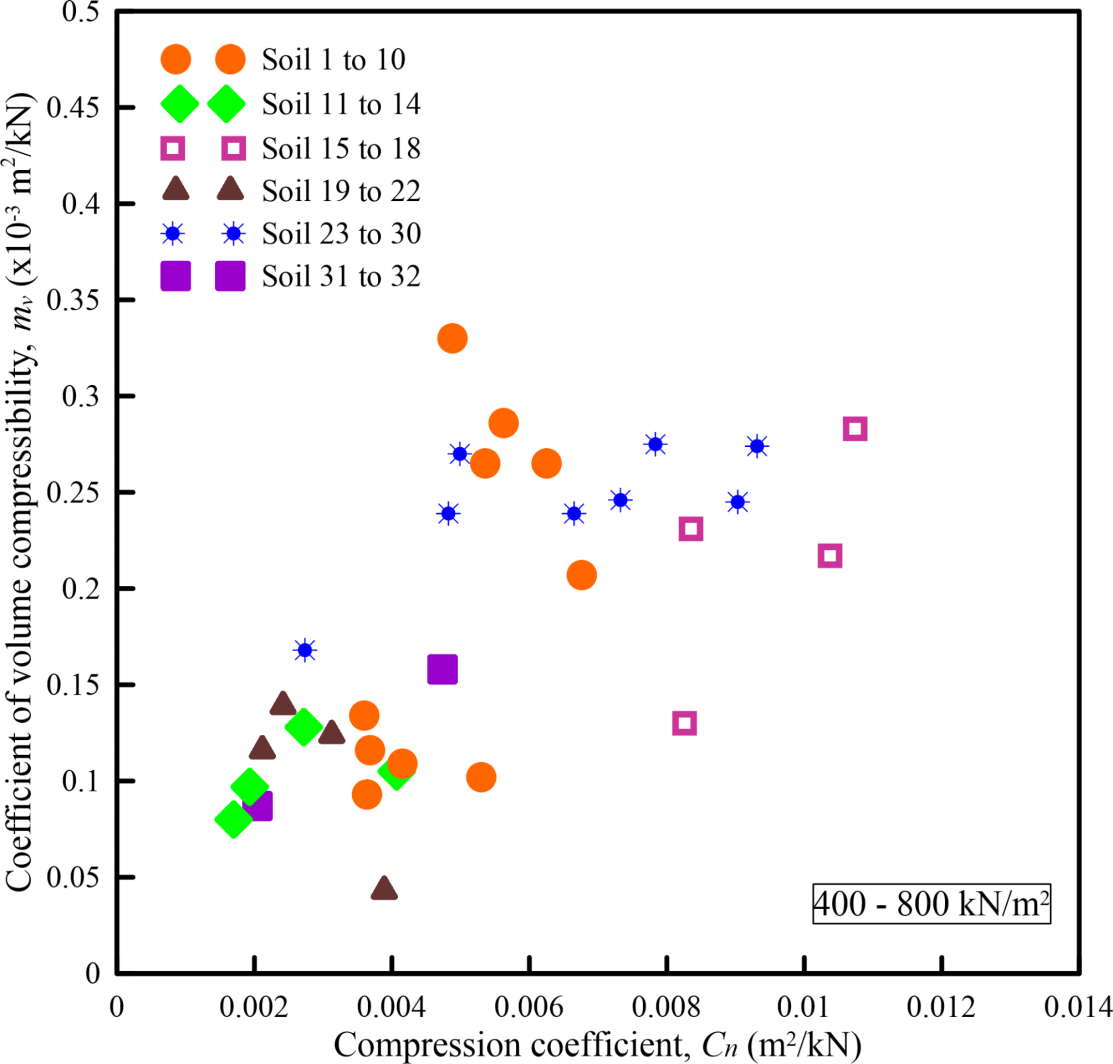
Variation of *m*_*v*_ with *C*_*n*_ for a pressure range of 400–800 kN/m^2^.

Figures 6, 7, 8, 9 and 10 show the variation of *m*_*v*_ with *C*_*n*_ for different pressure ranges. From these figures, it can be observed that though there exists an increasing relationship between *m*_*v*_ and *C*_*n*_, some amount of scatter can be observed. Further, these relationships are pressure-dependent and applicable only to those pressure ranges from which they were derived. From this observation, it is clear that, in addition to soil state and soil type, *m*_*v*_ is also dependent on the pressure increment and the initial pressure from which the pressure increment is computed. To overcome the above shortcoming, Parthasarathy ^10^, defined two non-dimensional parameters namely compression factor (*C*_*F*_) and normalized constrained modulus (*σ*_*0*_*/D*).

*C*_*F*_ (Eq. (3)) is the ratio of the product of *I*_*S*_ and incremental pressure (*Δσ*) to the *q*_*u*_ which will take care of the soil state, soil type, and the pressure increment.

Similarly, *σ*_*0*_*/D* (Eq. (4)), which is a product of *m*_*v*_ and initial effective overburden pressure (*σ*_*0*_) will take care of the range of pressure increment.

3 $${\rm Compression \, factor, }{C}_{F}=\:\left(\frac{\varDelta\:\sigma\:{I}_{S}}{{q}_{u}}\right)$$

4 $${\rm Normalized \, constrained \, modulus }\:=\frac{{\sigma\:}_{0}}{D}=\:{m}_{v}{\sigma\:}_{0}$$

**Fig. 11:**
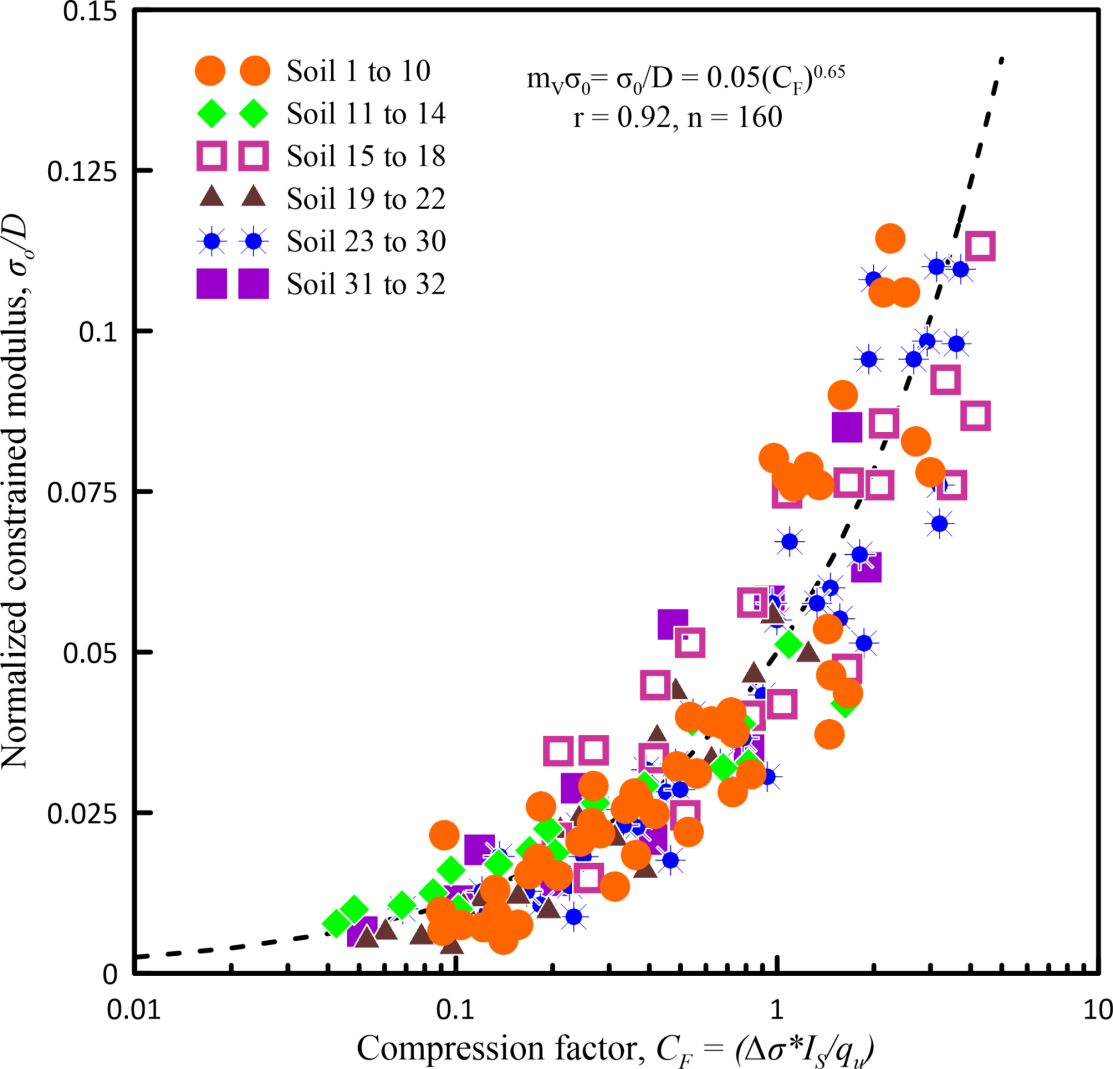
Variation of compression factor with normalized constrained modulus for all soil samples used in the present study.

Using these non-dimensional parameters, an attempt has been made to develop a correlation model to predict *m*_*v*_. Figure 11 shows a variation of compression factor with normalized constrained modulus from which it can be observed that there is a good non-linear relationship (Eq. (5)), from which *m*_*v*_ can be predicted.

5 $$\:\frac{{\sigma\:}_{0}}{D}=\:{m}_{v}{\sigma\:}_{0}=0.05{\left({C}_{F}\right)}^{0.65}$$

The relationship shown through Eq. (5) for predicting *m*_*v*_, overcomes the requirement of undisturbed soil sample required for evaluating both *q*_*u*_ and *m*_*v*_. It is a comprehensive model as it takes into account both soil state & soil type and is applicable for a wide range of pressures. Hence, it can be universally adopted for predicting *m*_*v*_ by practicing engineers and researchers.

## Conclusions

This study aims to develop a robust model for predicting the *m*_*v*_ by utilizing suitable parameters that represent both soil state and soil type. The significant conclusions drawn from this research are as follows:

- A strong correlation was observed between the standardized SPT *N*_60_ and *q*_*u*_, making SPT *N*_60_ a valuable parameter for representing soil state. This approach eliminates the necessity of obtaining undisturbed soil samples for assessing *q*_*u*_.
- It is observed that *I*_*S*_, which is the numerical difference between *w*_*L*_ and *w*_*S*_, effectively captures the range of volume change typically encountered in civil engineering applications., Thus, *I*_*S*_ serves as a superior parameter to represent soil type.
- By incorporating these two new parameters—SPT *N*_60_ for soil state and *I*_*S*_ for soil type—a correlation equation has been developed, as presented in Eq. (5), to predict *m*_*v*_. Additionally, since *m*_*v*_ is pressure-dependent, this characteristic parameter has also been integrated into the equation.
- Overall, the proposed model is comprehensive and can be universally applied, making it a valuable tool for practicing engineers and researchers in predicting *m*_*v*_ effectively.

## Data Availability

The datasets used and/or analyzed during the current study are available from the corresponding author upon reasonable request.

## List of symbols

*A*: Factor depending on initial overburden pressure (*P*_0_) and incremental stress (*ΔP*) (= 1.2 − 0.0015*P* and *P* = *P*_0_ + *ΔP*/2)
*c*_*c*_: Compression index
*C*_*F*_: Compression factor
*e*: Void ratio
*e*_0_: Initial void ratio
*f*_2_: Constant based on plasticity index
*I*_*P*_: Plasticity index
*I*_*S*_: Shrinkage index
*m*_*v*_: Coefficient of volume compressibility
*m*_*v*__0_: Standard m_v_ values based on water content
*N*_*LCPT*_: Resistance measured from light cone penetration test
*Nsw*: Number of half rotations in SWS test
*N*_60_: Standardized SPT N-value
*q*_*u*_: Unconfined compressive strength
*w*_*L*_: Liquid limit
*w*_*n*_: Natural water content
*w*_*P*_: Plastic limit
*w*_*S*_: Shrinkage limit
*Wsw*: Total weight of loads in Swedish Weight Sounding (SWS) test
α and *β*: Weighting factors depending on the number of half rotations and depth in SWS test
*σ*_0_: Initial effective overburden pressure
*σ*_0_*/D*: Normalized constrained modulus
*Δσ*: Incremental pressure
